# Plasma PCSK9 Levels Are Elevated with Acute Myocardial Infarction in Two Independent Retrospective Angiographic Studies

**DOI:** 10.1371/journal.pone.0106294

**Published:** 2014-09-02

**Authors:** Naif A. M. Almontashiri, Ragnar O. Vilmundarson, Nima Ghasemzadeh, Sonny Dandona, Robert Roberts, Arshed A. Quyyumi, Hsiao-Huei Chen, Alexandre F. R. Stewart

**Affiliations:** 1 Ruddy Canadian Cardiovascular Genetics Centre, University of Ottawa Heart Institute, Ottawa, Ontario, Canada; 2 Department of Biochemistry, Microbiology and Immunology, University of Ottawa, Ottawa, Ontario, Canada; 3 Center for Genetics and Inherited Diseases, Taibah University, Almadina, Saudi Arabia; 4 Department of Medicine, Emory University, Atlanta, Georgia, United States of America; 5 Department of Medicine, McGill University, Montreal, Canada; 6 Department of Medicine, University of Ottawa, Ottawa, Ontario, Canada; 7 Ottawa Hospital Research Institute, Ottawa, Ontario, Canada; Washington Hospital Center, United States of America

## Abstract

**Objective:**

Proprotein convertase subtilisin/kexin type 9 (PCSK9) is a circulating protein that promotes degradation of the low density lipoprotein (LDL) receptor. Mutations that block PCSK9 secretion reduce LDL-cholesterol and the incidence of myocardial infarction (MI). However, it remains unclear whether elevated plasma PCSK9 associates with coronary atherosclerosis (CAD) or more directly with rupture of the plaque causing MI.

**Methods and Results:**

Plasma PCSK9 was measured by ELISA in 645 angiographically defined controls (<30% coronary stenosis) and 3,273 cases of CAD (>50% stenosis in a major coronary artery) from the Ottawa Heart Genomics Study. Because lipid lowering medications elevated plasma PCSK9, confounding association with disease, only individuals not taking a lipid lowering medication were considered (279 controls and 492 with CAD). Replication was sought in 357 controls and 465 with CAD from the Emory Cardiology Biobank study. PCSK9 levels were not associated with CAD in Ottawa, but were elevated with CAD in Emory. Plasma PCSK9 levels were elevated in 45 cases with acute MI (363.5±140.0 ng/ml) compared to 398 CAD cases without MI (302.0±91.3 ng/ml, p = 0.004) in Ottawa. This finding was replicated in the Emory study in 74 cases of acute MI (445.0±171.7 ng/ml) compared to 273 CAD cases without MI (369.9±139.1 ng/ml, p = 3.7×10^−4^). Since PCSK9 levels were similar in CAD patients with or without a prior (non-acute) MI, our finding suggests that plasma PCSK9 is elevated either immediately prior to or at the time of MI.

**Conclusion:**

Plasma PCSK9 levels are increased with acute MI.

## Introduction

By promoting the degradation of low density lipoprotein (LDL) receptors (LDL-R) in the liver, proprotein convertase subtilisin/kexin type 9 (PCSK9) elevates plasma levels of LDL-cholesterol (LDL–C) [1]. In African Americans, loss-of-function mutations in PCSK9 that prevent its secretion are associated with a 30%–40% reduction in plasma levels of LDL-C, but surprisingly reduce events associated with coronary atherosclerosis (CAD, coronary artery disease) by 88% over a 15 year period [2]. A similar level of reduced LDL-C attained by statins would be associated with only a 25% reduction in the risk of coronary events. This observation has also been confirmed in individuals of European ancestry for the R46L PCSK9 variant. The MIGen consortium found markedly reduced risk of MI associated with this allele [3]. In another independent study, a large meta-analysis of several Danish cohorts found that carriers of the 46L allele had a 12% reduction in LDL-C that predicted a 5% reduction, but that was in fact associated with a 28% reduction, in ischemic heart disease [4]. These similar findings have been hypothesized to result from a lifetime reduction in LDL-C, reducing the burden of atherosclerosis, but could also be explained if PCSK9 does more than control LDL-R levels. Overall, plasma PCSK9 accounts for less than 8% of the variance of plasma LDL-C [5].

Statins, while lowering LDL-C, markedly elevate plasma PCSK9, most likely due to increased expression of the PCSK9 activating transcription factors SREBP2 and HNF1α [6]. It would appear that the LDL-C lowering effect of statins outweighs the risk associated with elevated PCSK9, because statins dose-dependently lower LDL-C in both men and women [7] and reduce cardiovascular events in women just as well as in men [8]. _ENREF_13_ENREF_14_ENREF_15Recent clinical trials using antibodies against PCSK9 that block PCSK9 interaction with the LDL-R were successful at lowering LDL-C below the levels obtained with statins over several weeks [9], [10] As a proof of concept, this approach has generated considerable excitement [11]. It remains to be seen whether therapies that lower plasma PCSK9 will lower the incidence of MI. Since MI occurs on a substrate of coronary atherosclerosis, whether elevated plasma PCSK9 increases MI risk through increased coronary atherosclerosis or by additional mechanisms remains unclear. Genetic studies have shown that variants contributing to the risk of coronary atherosclerosis can be distinguished from variants contributing to the risk of MI [12], [13]. If elevated plasma PCSK9 contributes to the risk of thrombosis and MI, rather than or in addition to the risk of atherosclerosis, then this could also account for the disproportionate reduction in MI risk associated with genetic variants that lower plasma PCSK9 levels. Importantly, if elevated plasma PCSK9 carries risk beyond its effect on LDL-C, there may be an additional benefit to lowering plasma PCSK9 in individuals treated with statins. Here, we asked whether elevated plasma PCSK9 is associated with MI or angiographically-defined CAD in two independent retrospective angiographic studies, the Ottawa Heart Genomics Study (OHGS) [14] and the Emory Cardiology Biobank (EmCB) study [15].

## Materials and Methods

### Study design

Plasma PCSK9 levels were measured by ELISA and tested for association with angiographic CAD and MI in two independent angiographic cross-sectional studies, the OHGS [14] with replication in a subset of the EmCB study [15].

### Study participants

For the OHGS, the Research Ethics Board of the University of Ottawa Heart Institute approved this study and written informed consent was obtained from all study participants. The OHGS excluded individuals with diabetes mellitus to ensure that the genetic risk primarily associated with coronary artery disease would not be confounded by the risk associated with diabetes, as described previously [16]. In addition, diabetes is known to elevate plasma PCSK9 levels [5], and a priori exclusion of diabetics in the OHGS study design removes this confounding variable.

CAD cases were identified by angiography as having a stenosis of greater than 50% in a major coronary artery, whereas angiographic controls had less than 30% stenosis. Severity of CAD was ranked according to the number of coronary arteries with stenosis, as described previously [16]. Patients were further stratified as having had an acute MI, according to elevated troponin and/or ST-elevation, if it was documented at the time of catheterization. Acute MI samples were obtained within 24–48 hours of the onset of symptoms, prior to the initiation of statin therapy. Otherwise, CAD cases were classified as having prior (or historical) MI or as having no MI from chart review. Controls were individuals who underwent coronary angiography prior to heart valve surgery or symptomatic individuals but with minimal (<30%) stenosis. Individuals with intermediate stenoses between 30% and 50% were excluded. Information regarding statin use, medication to treat hypertension, and recent (at the time of recruitment or within 12 months prior to recruitment) plasma LDL-C, HDL-C, and triglycerides was measured by colorimetric assays on a Roche Modular Analytics P Module clinical chemistry analyzer (Roche Diagnostics Inc., Laval, Quebec, Canada). Plasma samples were obtained at the time of cardiac catheterization from fasting individuals, stored at −80°C and thawed once for assessment of plasma PCSK9 levels. Of note, plasma samples were drawn at various times randomly distributed throughout the day.

The EmCB study was approved by the Institutional Review Board at Emory University, Atlanta, GA, USA. All subjects provided written informed consent at the time of enrollment. For the present study, 822 plasma samples were selected from 2,357 individuals of the EmCB study who underwent elective or emergent cardiac catheterization and who were not taking a statin or fibrate nor were diabetic at the time of recruitment. CAD cases were defined as having greater than 50% stenosis whereas controls had less than 20% stenosis, as described previously [15]. Similar criteria as in the OHGS were used to define CAD cases with acute, prior or no MI in the EmCB study.

### PCSK9 ELISA

Plasma levels of PCSK9 were measured by ELISA using a commercial assay (Quantikine ELISA, R&D Systems, Minneapolis, MN) that has been previously validated [17], [18]. Plasma samples were diluted 1∶20 in dilution buffer. The minimal detectable dose of PCSK9 was 0.1 ng/ml, with a typical range of 39 to 1,400 ng/ml in human plasma. We were concerned that plasma LDL-C might affect the measurement of plasma PCSK9 using the PCSK9 ELISA assay, since PCSK9 interacts with LDL-C [19]. The PCSK9 protein standard was diluted and incubated at 37°C for 30 minutes without or with the addition of LDL-C to a concentration of 2.6 mmol/L and no difference in PCSK9 levels was detected (data not shown). Thus, LDL-C does not interfere with the ELISA. PCSK9 levels in 12 individuals were tested in 6 replicates in one assay and again in a separate assay. Several different lots of the assay were used to measure PCSK9 in the OHGS samples, whereas a single lot was used for the EmCB study. The intra-assay coefficient of variance was 8.9% and between assays was 12%.

### Statistical analysis

Continuous variables are presented as mean ± standard deviation (SD) and categorical variables are presented as N (%). To determine whether differences between CAD cases and controls were significant for continuous and categorical variables the Student's *t*-test and χ^2^ test were used, respectively. To determine whether mean plasma PCSK9 levels differed between 0, 1, 2 or 3 vessel disease a one-way analysis of variance (ANOVA) was used. Multiple logistic regression analysis was used to examine whether PCSK9 was associated with CAD or acute MI when correcting for known risk factors (age, male sex, BMI, smoking, antihypertensive medication use, LDL-C, HDL-C, and TG). The acute MI variable compared CAD cases with no MI to those with acute MI, excluding CAD cases with prior MI except when an individual who had suffered a prior MI had also suffered an acute MI, in which case they were labeled as having an acute MI. Spearman's rank correlation was used to determine whether plasma PCSK9 and LDL-C levels are correlated. All statistical analyses were two-sided and performed with SPSS (version 19.0, SPSS Inc., Chicago IL, USA), SAS Enterprise Guide (v. 4.3, SAS Institute Inc., Cary NC, USA) and R (v. 3.0.2, The R Foundation for Statistical Computing, Vienna, Austria). One-way ANCOVA was used to determine whether PCSK9 levels differed between controls, CAD cases with no MI, CAD cases with acute MI and CAD cases with prior MI and post-hoc tests were adjusted for multiple comparisons by Sidak correction. The threshold for significance was set at p<0.05 for all analyses performed.

## Results

### Statins elevate plasma PCSK9

The clinical characteristics of the OHGS [14] samples are presented in Table 1, stratified by statin use because of the marked effect of statins to elevate plasma PCSK9 levels [6]. Plasma PCSK9 levels were not different between cases or controls in either those taking or not taking a statin. Linear regression analysis confirmed that plasma PCSK9 is elevated by statin use as well as by young age and female sex (Table 2). Smoking, antihypertensive medication and CAD were also independent predictors of PCSK9.

**Table 1:** Clinical characteristics of OHGS CAD cases and controls.

	Total			On Statin			Not taking statin		
Variable	Cases (N = 3273)	Controls (N = 645)	p	Cases (N = 2781)	Controls (N = 366)	p	Cases (N = 492)	Controls (N = 279)	p
Age, (years)	64±11	63±11	0.367	63±11	64± 11	0.665	65±11	63±12	0.003
Male sex, N (%)	2503 (76.5)	302 (46.8)	<0.001	2169 (78.0)	170 (46.4)	<0.001	334 (67.9)	132 (47.3)	<0.001
BMI, kg/m^^2^^	28.0±4.9	28.7±5.8	0.003	28.1±4.9	28.9±5.8	0.007	27.4±5.0	28.4±5.7	0.014
Smoking, N (%)	2354 (71.9)	374 (58.0)	<0.001	2009 (72.2)	233 (63.7)	<0.001	345 (70.1)	141 (50.5)	<0.001
HTN, N (%)	2029 (62.0)	514 (79.9)	<0.001	1784 (64.1)	307 (83.9)	<0.001	245 (49.8)	207 (74.2)	<0.001
Total-C, mmol/L	4.53±1.24	4.83±1.04	<0.001	4.43±1.22	4.64±1.03	0.002	5.26±1.08	5.15±0.98	0.246
LDL-C, mmol/L	2.61±1.05	2.79±0.87	<0.001	2.51±1.02	2.62±0.87	0.057	3.29±0.95	3.05±0.80	0.004
HDL-C, mmol/L	1.16±0.41	1.37±0.47	<0.001	1.15±0.41	1.32±0.44	<0.001	1.25±0.43	1.47±0.51	<0.001
TG, mmol/L	1.69±1.09	1.52±0.88	0.001	1.70±1.10	1.58±0.95	0.135	1.62±1.00	1.41±0.74	0.015
PCSK9, ng/mL	375.5±131.2	349.7±143.2	<0.001	387.3±132.6	374.6±146.5	0.117	309.0±99.7	317.1±131.9	0.376

Continuous variables presented as mean ± SD and categorical variables as N (%). Abbreviations: BMI, body mass index; HTN, anithypertensive medication, Total-C, total-cholesterol; LDL-C, Low density lipoprotein-cholesterol, HDL-C, high density lipoprotein-cholesterol, TG, triglycerides.

**Table 2:** Linear regression analysis of the relationships between explanatory variables and the level of PCSK9 in the OHGS.

Variable	β	p
Age, (years)	−0.086	<0.001
Male sex	−0.151	<0.001
BMI, kg/m^2^	0.024	0.127
Smoking	0.033	0.033
HTN	−0.050	0.002
Statin (including fibrates)	0.225	<0.001
CAD	0.041	0.013

Abbreviations: BMI, body mass index; HTN, anithypertensive medication. N = 3,918.

### Plasma PCSK9 levels are not associated with coronary atherosclerosis in the OHGS for individuals not taking a statin

**Figure 1:**
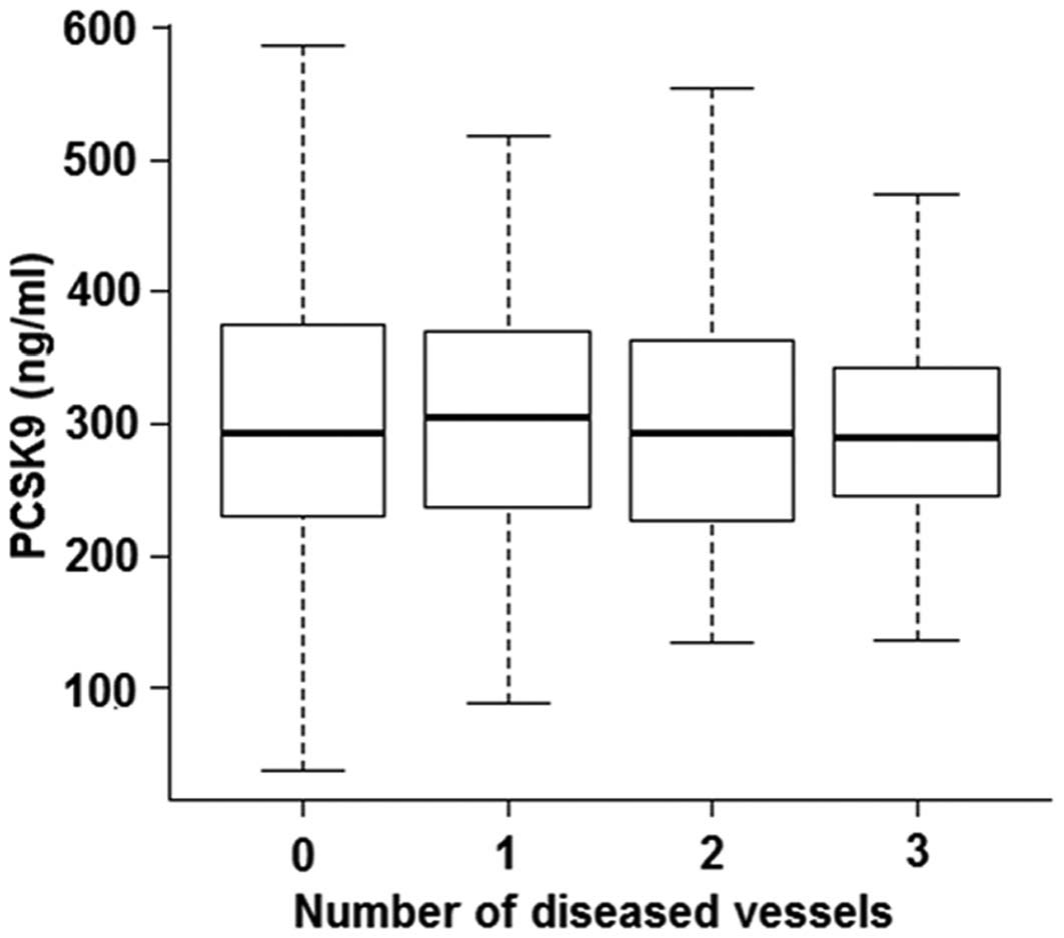
Mean PCSK9 levels do not differ with atherosclerosis burden. A Tukey's boxplot displaying PCSK9 levels in OHGS CAD controls (0 diseased vessels) and cases (1, 2 or 3 diseased vessels) in individuals not taking statins. The median is the line in the box, the 1^st^ and 3^rd^ quartiles are the upper and lower edges of the boxes and 1.5 interquartile range (IQR) is displayed as whiskers. Outliers have been removed. (0 vessel disease, N = 280; 1-vessel disease, N = 126; 2-vessel disease, N = 102; 3-vessel disease, N = 104).

To determine whether PCSK9 levels are associated with CAD, we performed a logistic regression analysis correcting for known CAD risk factors (Table 3). However, given the known marked effect of statins [6], [20] and fibrates [21], [22] to elevate PCSK9 levels, also seen in our study (Table 2), we stratified the analysis by statin use. PCSK9 levels were only associated with CAD in individuals taking a statin (Table 3, p = 0.0003), suggesting that the observed effect of CAD on PCSK9 levels (Table 2) is driven by higher statin use among CAD cases. Similarly, no association between PCSK9 levels and severity of CAD, assessed by the number of diseased vessels, was observed in the OHGS by ANOVA in individuals not taking a statin (Figure 1). Given that plasma PCSK9 levels did not associate with CAD in individuals not taking a statin, we asked whether plasma PCSK9 levels might instead be associated with acute or prior MI.

**Table 3:** Logistic regression analysis of the association of PCSK9 with CAD in the OHGS cohort, stratified by statin use.

	On statin		Not taking statin	
Variable	β or OR (95% CI)	p	β or OR (95% CI)	p
Age, (years)	0.004 (−0.009, 0.018)	0.530	0.013 (−0.007, 0.034)	0.210
Male sex	2.776 (2.075, 3.714)	6.10E-12	1.382 (0.875, 2.184)	0.166
BMI, kg/m^2^	−0.033 (−0.059, −0.006)	0.015	−0.035 (−0.076, 0.006)	0.091
Smoking	1.261 (0.946, 1.68)	0.113	3.343 (2.156, 5.184)	7.00E-08
HTN	0.379 (0.267, 0.538)	5.92E-08	0.442 (0.282, 0.694)	3.88E-04
LDL, mmol/L	−0.125 (−0.259, 0.009)	0.068	0.251 (−0.004, 0.506)	0.053
HDL, mmol/L	−0.612 (−0.916, −0.308)	7.86E-05	−0.887 (−1.396, −0.378)	0.001
TG, mmol/L	0.366 (−0.319, 1.050)	0.295	0.733 (−0.331, 1.797)	0.177
PCSK9, ng/mL	0.002 (0.001, 0.003)	0.0004	−0.001 (−0.002, 0.001)	0.505

Results given as beta for continuous variables and odds ratio (OR) for categorical variables. Abbreviations: BMI, body mass index; HTN, anithypertensive medication; Total-C, total-cholesterol; LDL-C, Low density lipoprotein-cholesterol; HDL-C, high density lipoprotein-cholesterol; TG, triglycerides. On statin, N = 2,396; Not taking statin, N = 478. Samples with missing values for variables were excluded.

### Plasma PCSK9 levels are elevated with acute MI, but not with prior MI, in the OHGS

**Figure 2:**
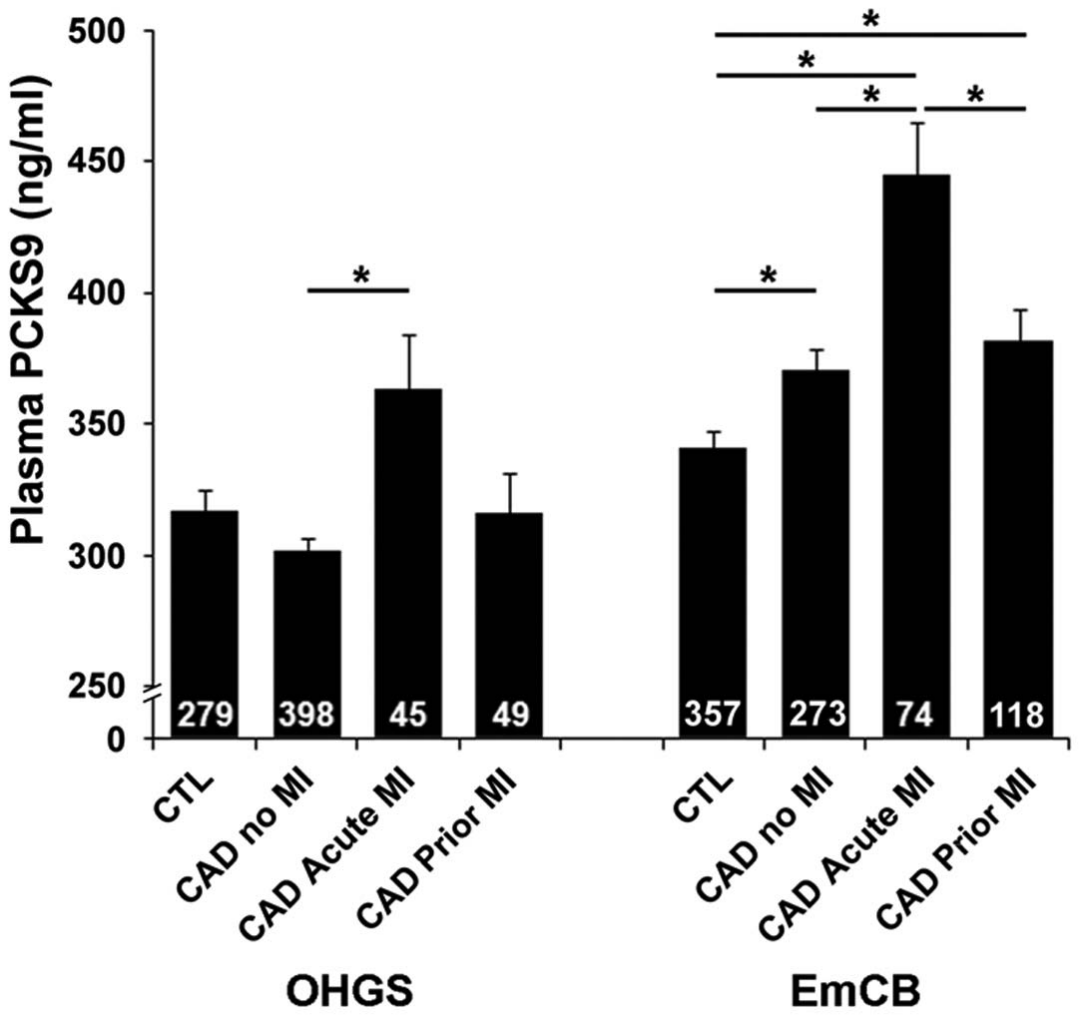
Plasma PCSK9 levels are increased with acute MI. Numbers in columns reflect sample size per group for individuals not taking a lipid-lowering medication (statin or fibrate) at the time of recruitment. Values are mean±SEM. Asterisks indicate significantly elevated PCSK9 by ANCOVA, p<0.05 after adjusting for variables (age, male sex, BMI, antihypertensive medication use, and smoking) and correcting for multiple comparisons.

Out of the 3,273 CAD patients in the OHGS, 1,371 had an MI (799 with prior history of MI and 572 with acute MI), and of those with an MI, 94 were not taking a statin (6.8%); 45 had acute MI and 49 had prior MI. Plasma PCSK9 levels were markedly elevated in 45 individuals with acute MI (363.5±140.0 ng/ml) compared to 398 CAD cases without MI (302.0±91.3 ng/ml, p = 0.004), adjusted for age at consent, male sex, BMI, smoking, and antihypertensive meds (Figure 2). In contrast, no difference in PCSK9 levels was seen between 49 individuals with a prior MI (315.9±107.5 ng/ml) and 398 CAD cases without an MI (p = 0.977). A mean of 11.4 ± 8.1 years separated the occurrence of MI from the time of recruitment in this group. Elevated PCSK9 levels seen with acute MI were not accompanied by elevated LDL-C levels (3.051±0.090 mmol/L for acute MI, 3.322±0.914 mmol/L for CAD no MI, p = 0.12). Logistic regression analysis confirmed that PCSK9 levels are an independent predictor of acute MI in CAD cases not taking a statin in the OHGS (Table 4).

**Table 4:** Logistic regression analysis of the association of PCSK9 with acute MI in the OHGS cohort, stratified by statin use.

	On statin		Not taking statin	
Variable	β or OR (95% CI)	p	β or OR (95% CI)	p
Age, (years)	−0.024 (−0.036, −0.012)	4.79E-05	−0.013 (−0.052, 0.027)	0.523
Male sex	0.876 (0.655, 1.172)	0.374	0.692 (0.268, 1.786)	0.447
BMI, kg/m^2^	−0.039 (−0.065, −0.013)	0.003	0.015 (−0.063, 0.093)	0.705
Smoking	1.287 (0.9997, 1.658)	0.050	0.94 (0.369, 2.391)	0.896
HTN	0.752 (0.594, 0.953)	0.019	0.643 (0.277, 1.49)	0.303
LDL, mmol/L	−0.049 (−0.163, 0.065)	0.403	−0.346 (−0.836, 0.143)	0.166
HDL, mmol/L	−1.068 (−1.470, −0.665)	1.97E-07	−2.038 (−3.455, −0.622)	0.005
TG, mmol/L	−0.139 (−0.751, 0.472)	0.655	−1.093 (−3.143, 0.956)	0.296
PCSK9, ng/ml	0.0004 (−0.0004, 0.001)	0.307	0.005 (0.002, 0.009)	0.002

Results given as beta for continuous variables and odds ratio (OR) for categorical variables. Abbreviations: BMI, body mass index; HTN, anithypertensive medication; Total-C, total-cholesterol; LDL-C, Low density lipoprotein-cholesterol; HDL-C, high density lipoprotein-cholesterol; TG, triglycerides. On statin, N = 1,568; Not taking statin, N = 287.

### PCSK9 levels are associated with coronary atherosclerosis in the EmCB study

We next sought “a priori” confirmation of our findings in an independent cohort. The Emory Cardiology Biobank (EmCB) study is another large angiographic cohort consisting of more than 2,400 individuals [15]. To avoid the confounding effect of statins and to replicate the selection criteria of the OHGS cohort (non-diabetic), we measured plasma PCSK9 levels in 465 CAD cases and 357 controls without diabetes mellitus and not taking a statin or fibrate at the time of recruitment (see Table 5). Linear regression analysis also showed that CAD was significantly associated with elevated plasma PCSK9 (Table 6). In contrast to the subset of individuals not taking a statin or fibrate in the OHGS, plasma PCSK9 levels were elevated in angiographic CAD cases (385.0±146.9 ng/ml) compared to controls (340.4±125.2 ng/ml, p<0.001) in the EmCB sample. Logistic regression confirmed that plasma PCSK9 levels are an independent predictor of CAD in the EmCB sub-study (Table 7). The overall higher PCSK9 values in the EmCB study likely reflect a lot-specific variation in the PCSK9 ELISA.

**Table 5:** Clinical characteristics of CAD cases and controls from the EmCB sub-study.

Variable	Cases (N = 465)	Controls (N = 357)	p
Age, (years)	65±12	56±12	<0.001
Male sex, N (%)	352(75.7)	193 (54.1)	<0.001
BMI, kg/m^^2^^	28.3±5.5	29.8±6.8	<0.001
Smoking, N (%)	92 (19.8)	48(13.4)	0.01
HTN, N (%)	327(70.3)	201 (56.3)	<0.001
Total-C, mmol/L	4.52±1.22	4.73±1.13	0.028
LDL-C, mmol/L	2.73±1.05	2.86±0.88	0.070
HDL-C, mmol/L	1.07±0.34	1.15±0.37	0.005
TG, mmol/L	1.68±1.27	1.54±1.05	0.140
PCSK9, ng/mL	385.0±146.9	340.4±125.2	0.000003

All EmCB samples were from non-diabetic individuals not taking a statin or fibrate at the time of recruitment. Continuous variables presented as mean ± SD and categorical variables as N (%). Abbreviations: BMI, body mass index; HTN, anithypertensive medication; Total-C, total-cholesterol; LDL-C, Low density lipoprotein-cholesterol; HDL-C, high density lipoprotein-cholesterol; TG, triglycerides.

**Table 6:** Linear regression analysis of the relationships between explanatory variables and the level of PCSK9 in the EmCB sub-study.

Variable	β	P-value
Age, (years)	−0.077	0.054
Male sex	−0.010	0.775
BMI, kg/m^2^	−0.015	0.686
Smoking	−0.034	0.348
HTN	−0.058	0.103
CAD	0.199	<0.001

Abbreviations: BMI, body mass index; HTN, anithypertensive medication. N = 822.

**Table 7:** Logistic regression analysis of the association of PCSK9 with CAD in the EmCB sub-study.

Variable	β or OR (95% CI)	p
Age, (years)	−0.005 (−0.034, 0.024)	<2E-16
Male sex	3.408 (2.28, 5.094)	2.24E-09
BMI, kg/m^2^	0.034 (−0.019, 0.088)	0.103
Smoking	2.774 (1.654, 4.651)	0.0001
HTN	2.002 (1.368, 2.929)	0.0003
LDL-C, mmol/L	0.003 (−0.003, 0.010)	0.235
HDL-C, mmol/L	−0.018 (−0.044, 0.008)	0.089
TG, mmol/L	−0.438 (−1.716, 0.841)	0.256
PCSK9, ng/mL	0.003 (0.001, 0.005)	5.26E-06

All EmCB samples were from non-diabetic individuals not taking a statin at the time of recruitment. Results given as beta for continuous variables and odds ratio (OR) for categorical variables. Abbreviations: BMI, body mass index; HTN, anithypertensive medication; Total-C, total-cholesterol; LDL-C, Low density lipoprotein-cholesterol; HDL-C, high density lipoprotein-cholesterol; TG, triglycerides. N = 677.

### Plasma PCSK9 levels are also elevated with acute MI, but not with prior MI, in the EmCB study

Similar to the OHGS sample, plasma PCSK9 levels were elevated in the 74 individuals with acute MI (445.0±171.7 ng/mL) compared to the 273 individuals with CAD but no MI (369.9±139.1 ng/mL, p = 3.7×10^−4^, after adjusting for variables, Figure 2). Here too, elevated plasma PCSK9 levels with acute MI were not associated with LDL-C levels (2.94±1.28 mmol/L for acute MI and 2.75±1.00 mmol/L for CAD no MI, p = 0.423). Also similar to the OHGS, no difference was seen between the 118 individuals with prior MI (382.3±139.1 ng/mL) and 273 CAD cases without MI (369.9±139.13 ng/mL, p = 0.0.954). An average of 10.1 ± 9.3 years elapsed between the MI and the time of recruitment for CAD cases of prior MI in the EmCB study. Logistic regression analysis confirmed that PCSK9 levels are an independent predictor of acute MI in CAD cases in the EmCB sub-study (Table 8).

**Table 8:** Logistic regression analysis of the association of PCSK9 with acute MI in the EmCB sub-study.

Variable	β or OR (95% CI)	p
Age, (years)	−0.005 (−0.034, 0.024)	0.718
Male sex	0.745 (0.402, 1.378)	0.200
BMI, kg/m^2^	0.034 (−0.019, 0.088)	0.209
Smoking	1.127 (0.576, 2.207)	0.542
HTN	0.627 (0.358, 1.097)	0.370
LDL, mmol/L	0.003 (−0.003, 0.010)	0.316
HDL, mmol/L	−0.018 (−0.044, 0.008)	0.182
TG, mmol/L	−0.438 (−1.716, 0.841)	0.502
PCSK9, ng/mL	0.003 (0.001, 0.005)	0.0008

Results given as beta for continuous variables and odds ratio (OR) for categorical variables. Abbreviations: BMI, body mass index; HTN, anithypertensive medication; Total-C, total-cholesterol; LDL-C, Low density lipoprotein-cholesterol; HDL-C, high density lipoprotein-cholesterol; TG, triglycerides. N = 296.

In individuals taking statins, there was no correlation between PCSK9 levels and plasma LDL-C (r_s_ =  −0.0288, p = 0.158, N = 2,410) in the OHGS. In individuals not taking statins, there was also no correlation between PCSK9 levels and LDL cholesterol (r_s_ = 0.0813, p = 0.0754, N = 479). When the analysis is stratified by CAD, a weak positive correlation between the levels of PCSK9 and LDL cholesterol (r_s_ = 0.176, p = 0.0257, N = 160) was observed in controls not taking statins. No correlation was seen in CAD cases not taking statins, nor in CAD cases (r_s_ = −0.0268, p = 0.214, N = 2155) or controls (r_s_ = −0.0114, p = 0.856, N = 225) taking statins. Similarly, in the EmCB sub-study of individuals not taking statins, there was no correlation between PCSK9 levels and LDL cholesterol in either those with CAD (r_s_ = −0.00840, p = 0.862, N = 429) or controls (r_s_ = −0.00742, p = 0.895, N = 322).

## Discussion

Here, for the first time to our knowledge, we found that plasma PCSK9 is elevated with acute MI in non-diabetic individuals who are not taking a statin and have angiographically proven CAD. A strength of our study is that this finding was replicated in a second independent cohort, an EmCB sub-study. Furthermore, our observation that plasma PCSK9 levels are not elevated in individuals with prior MI compared to those with angiographic CAD that did not have an MI argues that PCSK9 levels are transiently elevated with acute MI.

Since lipid lowering medications elevate plasma PCSK9 [6], [20], [22]_ENREF_20, it is necessary to exclude individuals on lipid lowering medications to avoid possible spurious associations due to medication. However, given the high prevalence of cholesterol-lowering prescriptions in older Americans [23], as high as 44.9% in persons over age 60, it is a challenge to obtain a large sample size. Nevertheless, we were able to identify 45 individuals in the OHGS and 74 in the EmCB study with acute MI that were not taking a lipid-lowering medication at the time of recruitment. It is likely that individuals with CAD and prior MI not taking a statin may have been intolerant to statins, since statin-induced myopathy is estimated to affect 10–15% of statin users [24].

Our findings are consistent with genetic studies that have demonstrated a causal link between lower plasma PCSK9 levels and reduced risk of MI [2]–[4], [25]. The mechanism whereby PCSK9 contributes to the risk of MI has been attributed largely to its effect on degrading the LDL-R, thereby elevating LDL-C and promoting atherosclerosis. Indeed, MI occurs on a foundation of coronary atherosclerosis, and most genetic factors that contribute to the elevation of coronary atherosclerosis risk were discovered through their association with the increased risk of MI [26]. However, our finding that elevated plasma PCSK9 is associated with acute MI, rather than prior MI or with coronary atherosclerosis cautions that the relationship of PCSK9 to MI risk may be more complex than previously thought.

It is well recognized that elevated LDL-C is one of the most significant risk factors of atherosclerosis. Intriguingly, among the top 10 genetic variants associated with elevated LDL-C, a PCSK9 variant had the weakest association with elevated LDL-C but had the third strongest effect on the risk of MI [27]. In other words, the effect of PCSK9 on MI risk far exceeds its effect on plasma LDL-C levels and on coronary atherosclerosis risk. It is noteworthy that in the present study, in individuals not taking a statin, only a weak (OHGS) or no correlation was seen between plasma PCSK9 levels and LDL-C.

PCSK9 might increase the risk of MI by a mechanism distinct from its well-known effect on LDL–C. We observed a weak correlation between plasma PCSK9 levels and LDL-C only in controls not taking a lipid lowering medication in the OHGS but not in the EmCB study. In contrast to the increased levels of PCSK9 we observed in acute MI patients (measured 1 to 2 days after MI), plasma levels of LDL-C have been reported to fall transiently following MI [28]–[30]. Thus, PCSK9 levels are uncoupled from LDL-C, at least at the time of MI. One possibility is that PCSK9 levels increase transiently after the trauma of an MI, as an acute phase reactant. However, a recent study reported that plasma PCSK9 levels were not elevated at the time of admission for severe trauma, but only eight days after a traumatic injury and correlated with the severity of the trauma [31], arguing that PCSK9 is not an acute phase reactant.

Another intriguing possibility is that PCSK9 levels might be elevated prior to acute MI, and even trigger MI. PCSK9 has been reported to bind to the LDL-R related protein LRP8 [32]. LRP8 activation promotes platelet aggregation [33]. If PCSK9 binding activates LRP8 receptor signaling, it could increase platelet aggregation and augment the risk of MI. Acute coronary events are more frequent in the morning [34], at a time when plasma PCSK9 levels are at their highest [35], and when propensity for platelets to aggregate is also increased [36]. Whether PCSK9 contributes to the risk of MI through platelet activation remains to be tested. In addition, in the OHGS sample, smoking was an independent predictor of circulating PCSK9 levels. This effect has been reported previously in a study of 4 healthy cohorts from Sweden comprising 5,722 middle-aged individuals [37], and although the mechanism underlying this association has not been explored, smoking is the second most important modifiable risk factor for MI after elevated LDL-C [38].

Plasma PCSK9 levels are elevated in patients with periodontitis, independently of LDL-C levels [17]. Periodontitis is a known risk factor for CAD and MI [39]. The risk of MI is elevated fivefold after an acute infection [40]. In mice, endotoxin elevates plasma PCSK9 levels [41] and plasma PCSK9 levels might be similarly elevated in humans after an acute infection and contribute to increased risk of subsequent MI. PCSK9-targeted therapies might lower the risk of events after acute infections. More needs to be learned about the functions of PCSK9 beyond its effect on LDL-R degradation.

One limitation of our study is that plasma samples from the OHGS were not carried forward to control for lot-to-lot variability when the EmCB samples were assayed. Thus, the 20% higher values measured for the EmCB samples could reflect a true difference or more likely reflect a lot-to-lot difference. Another limitation of our study is that both the OHGS and the EmCB are cross-sectional rather than longitudinal studies, having recruited angiographically-defined CAD cases and controls at the time of cardiac catheterization. Thus, it remains to be determined whether plasma PCSK9 levels are elevated as a consequence of acute MI (as an acute phase reactant) or prior to MI. In addition, we do not know why PCSK9 levels were not associated with CAD in those not taking statin in the OHGS, but were in the EmCB study. This may be a reflection of the presence of less coronary atherosclerosis in EmCB angiographic controls, where 20% or less stenosis was used as an inclusion criterion compared to 30% or less for the OHGS. Another factor could be the inclusion of individuals with aortic valve disease, who showed minimal coronary stenosis, in the OHGS controls [14]. It is not known whether aortic valve disease elevates plasma PCSK9 levels. A recent study of 243 angiographic cases reported a correlation between the severity of coronary artery disease and plasma PCSK9 levels [42]. However, in two larger cohorts, we did not replicate this finding.

## Conclusion

Among non-diabetic individuals with angiographically-defined coronary artery disease who are not taking a lipid-lowering medication, plasma PCSK9 levels are elevated at the time of acute myocardial infarction. Future longitudinal studies will be required to determine whether PCSK9 levels are elevated prior to or as a consequence of acute coronary events.
